# The evolutionary history of Stomatopoda (Crustacea: Malacostraca) inferred from molecular data

**DOI:** 10.7717/peerj.3844

**Published:** 2017-09-21

**Authors:** Cara Van Der Wal, Shane T. Ahyong, Simon Y.W. Ho, Nathan Lo

**Affiliations:** 1 School of Life and Environmental Sciences, University of Sydney, Sydney, Australia; 2 Australian Museum, Sydney, Australia; 3 School of Biological, Earth and Environmental Sciences, University of New South Wales, Kensington, Australia

**Keywords:** Stomatopoda, Phylogenetic analysis, Molecular clock, Fossil calibration, Ancestral state reconstruction

## Abstract

The crustacean order Stomatopoda comprises seven superfamilies of mantis shrimps, found in coastal waters of the tropics and subtropics. These marine carnivores bear notable raptorial appendages for smashing or spearing prey. We investigated the evolutionary relationships among stomatopods using phylogenetic analyses of three mitochondrial and two nuclear markers. Our analyses recovered the superfamily Gonodactyloidea as polyphyletic, with *Hemisquilla* as the sister group to all other extant stomatopods. A relaxed molecular clock, calibrated by seven fossil-based age constraints, was used to date the origin and major diversification events of stomatopods. Our estimates suggest that crown-group stomatopods (Unipeltata) diverged from their closest crustacean relatives about 340 Ma (95% CRI [401–313 Ma]). We found that the specialized smashing appendage arose after the spearing appendage ∼126 Ma (95% CRI [174–87 Ma]). Ancestral state reconstructions revealed that the most recent common ancestor of extant stomatopods had eyes with six midband rows of hexagonal ommatidia. Hexagonal ommatidia are interpreted as plesiomorphic in stomatopods, and this is consistent with the malacostracan ground-plan. Our study provides insight into the evolutionary timescale and systematics of Stomatopoda, although further work is required to resolve with confidence the phylogenetic relationships among its superfamilies.

## Introduction

Stomatopoda is one of the most distinctive orders of Crustacea. Commonly known as mantis shrimps, stomatopods are benthic, marine carnivores that are common in tropical and subtropical coastal waters (Schram et al., 2013). They are among the most efficient crustacean predators, having unique adaptations for hunting (Ahyong & Jarman, 2009). These adaptations include the second maxilliped modified as a powerful raptorial claw. The form of the raptorial claw and nature of the strike distinguishes two major functional groups, ‘smashers’ and ‘spearers’ (Patek, Korff & Caldwell, 2004; Patek & Caldwell, 2005). Although all stomatopods can both smash and spear prey depending on whether the dactyl is folded or unfolded, most have a raptorial claw that is highly optimized to either spear or to smash. In particular, specialized spearers have the dactylus of the raptorial claw lined with serrated spines and elongated raptorial claw segments enabling significant reach and prey retention. The raptorial claws of specialized smashers are optimized for impact by having a heavily calcified heel on the dactylus, and greatly enhanced meral musculature to drive a more powerful strike. In addition to their powerful raptorial apparatus, adult stomatopods have remarkable compound eyes (Marshall, Cronin & Kleinlogel, 2007). The ‘conventional’ larval cornea is replaced at the post-larval stage with a tripartite cornea divided into upper and lower halves separated by a midband of ommatidia containing elements capable of detecting polarized light and, in many groups, colour (Ahyong, 2005; Kleinlogel & Marshall, 2006; Chiou et al., 2008; Porter et al., 2010). Numbering almost 500 species, mantis shrimps play important roles in numerous marine ecosystems, owing to their large biomass and trophic position as both predator and prey (Geary, Allmon & Reaka-Kudla, 1991; Ahyong, Charbonnier & Garassino, 2013).

Stomatopoda comprises three suborders, Palaeostomatopodea (Devonian–Carboniferous), Archaeostomatopodea (Carboniferous), and Unipeltata (Jurassic–Recent), of which the latter two are most closely related (Schram, 2007; Haug et al., 2010). All extant stomatopods belong to the suborder Unipeltata, which comprises seven extant superfamilies, 17 families, and over 100 genera, most of which are contained in the superfamilies Gonodactyloidea, Lysiosquilloidea, and Squilloidea (Ahyong & Harling, 2000; Ahyong & Jarman, 2009). Each superfamily has a distinctive morphology and ecology, with recognizable differences in their raptorial appendage, visual systems, colour patterns, and telson ornamentation (Ahyong & Harling, 2000; Ahyong, 2005). Many such differences relate to habitat, particularly environmental light conditions, substrate type, and shelter availability (Ahyong, 1997; Porter et al., 2010).

Although vision, development, social behaviour, and alpha taxonomy of stomatopods have been studied in some detail (Caldwell, 1991; Manning, 1995; Froglia, 1996), few attempts have been made to infer the phylogeny of the group until the last two decades (Ahyong, 1997; Hof, 1998; Ahyong & Harling, 2000; Barber & Erdmann, 2000; Ahyong & Jarman, 2009; Porter et al., 2010). Recently, most extensive estimates of the stomatopod phylogeny have been based strictly on morphological characters, with few molecular phylogenetic studies. Consequently, the relationships among and within the seven superfamilies have not been extensively tested, and the evolutionary origins of their remarkable characters are not fully understood.

Molecular phylogenetic analyses of stomatopods were first conducted by Barber & Erdmann (2000), focusing on the superfamily Gonodactyloidea, which is dominated by smashing stomatopods. Ahyong & Jarman (2009) were the first to analyse molecular data from multiple superfamilies, and showed that specialized smashing evolved only once, possibly from a simplified spearing form. However, their study included representatives of only three of the seven superfamilies. Porter et al. (2010) included sequence data from a fourth superfamily, and examined the evolution of the visual system. They also found a single evolutionary origin of specialized smashing, and that many of the modern superfamilies are likely to have lost morphological complexity in their visual systems, particularly when compared with Gonodactyloidea. However, both of these molecular studies found low support for their estimates of the deep relationships among stomatopods (Ahyong & Jarman, 2009; Porter et al., 2010).

Uncertainty surrounds key aspects of stomatopod evolution, including the timing of the evolution of specialized raptorial spearing and smashing. Stomatopoda has a relatively diverse Paleozoic fossil record, with Archaeostomatopodea dating from the Carboniferous (∼313 Ma) in *Daidal acanthocercus* (see Schram, 2007). The Mesozoic and Cenozoic record, restricted to Unipeltata, is relatively sparse, particularly when compared with that of the Decapoda (Ahyong, 1997; Hof, 1998; Ahyong, 2005; Haug et al., 2013). The Mesozoic fossil record includes the Jurassic and Cretaceous stem unipeltatans (Sculdidae and Pseudosculdidae), along with representatives of some of the crown-group superfamilies (Lysiosquilloidea, Gonodactyloidea, and Squilloidea), suggesting that the major superfamilies diverged in the Cretaceous (Ahyong & Jarman, 2009). A large portion of the known fossil record is attributed to only a few locations, mainly in Europe, the Middle East, and North America, reflecting the geographic positions of tropical marine continental margins during the Mesozoic and Cenozoic. As with extant stomatopods, squilloids dominate the fossil record (Ahyong, Charbonnier & Garassino, 2013; Schram et al., 2013).

In this study, we use a molecular phylogenetic approach to investigate the evolutionary history of unipeltatan stomatopods. We present a comprehensive estimate of the phylogenetic relationships in the order, based on sequence data from six of its seven extant superfamilies. Using a Bayesian relaxed molecular clock, we estimate the evolutionary timeframe for the diversification of the group and the appearance of its remarkable raptorial appendage and visual systems. Our results shed light on past events that might have influenced the diversification of stomatopods, and test previous morphology-based phylogenetic hypotheses.

## Methods

### Taxon sampling and DNA sequencing

Thirty-eight stomatopod species, representing six superfamilies, 12 families, and 28 genera, were included in this study (Table S1). Sequences were either generated *de novo* or obtained from GenBank. Sequences obtained from GenBank were collected from single specimens in most cases; otherwise they were from multiple specimens collected from the same location. Tissue samples were collected from specimens provided by the Australian Museum (AM) and Muséum National d’Histoire Naturelle Paris (MNHN). Outgroup taxa were selected on the basis of previous analyses of stomatopods, sequence availability, and uncertainties over the phylogenetic position of Hoplocarida within Malacostraca (Richter & Scholtz, 2001; Miller & Austin, 2006; Ahyong & Jarman, 2009; Liu & Cui, 2010). Representatives of five other malacostracan lineages were included, representing Decapoda (*Homarus americanus* and *Homarus gammarus*), Anaspidacea (*Anaspides tasmaniae*), Euphausiacea (*Meganyctiphanes norvegica*), Peracarida (*Neomysis americana* and *Neomysis integer*), and Phyllocarida (*Paranebalia longipes*). GenBank accession numbers for all sequences are given in Table S1.

DNA was extracted using a modified version of the Chelex rapid-boiling procedure (Walsh, Metzger & Higuchi, 1991; Ahyong & Jarman, 2009). Three molecular markers were selected for amplification, based on the study by Ahyong & Jarman (2009). Regions of two mitochondrial genes (*12S* and *16S*) and one nuclear gene (D1 region of *28S*) were amplified using three sets of primers (Table S2). PCR cycle conditions differed for each primer set. The *12S* cycle parameters followed those of Mokady et al. (1994) and Mokady & Brickner (2001). The *16S* cycle parameters followed Ahyong & Jarman (2009), whereas the *28S* cycle parameters followed Schnabel, Ahyong & Maas (2011).

Sanger sequencing was performed by Macrogen (Seoul, South Korea). Contigs were aligned using the default assembly parameters in Sequencher v5.0.1 (Gene Codes Corporation). These data were combined with published sequences for *16S*, *18S*, cytochrome *c* oxidase subunit 1 (*CO1)*, and *28S* (D2–D7b and D9–D10 regions). Sequences were aligned separately for each gene using MUSCLE v3.8.31 (Edgar, 2004), with poorly aligned sites removed following visual inspection. Additionally, owing to large inconsistencies with the sequences from other taxa, we excluded small sections of the *28S* sequences from *Squilla rugosa* (sites 3,737–3,895) and *Kempella mikado* (sites 3,856–3,911). Sequence alignments are available online (https://github.com/caravanderwal/Stomatopoda).

To check for saturation of nucleotide substitutions, we analysed each gene separately using Xia’s test in DAMBE v6 (Xia, 2013). We also performed separate tests of saturation for each of the three codon positions in *CO1*. We found evidence of saturation at the third codon positions in *CO1*, but not in any other subsets of the data (Table S3). Therefore, we excluded the third codon sites of *CO1* from our phylogenetic analyses.

### Phylogenetic analysis

We performed phylogenetic analyses of our data set using both maximum likelihood and Bayesian inference. The best-fitting data-partitioning scheme and substitution models were selected using PartitionFinder v1.1.1 (Lanfear et al., 2012). The optimal partitioning scheme split the data into four subsets: (i) *12S* and *16S*; (ii) *18S*; (iii) first and second codon positions of *CO1*; and (iv) *28S*. For these four subsets, the best-fitting substitution models were GTR + G, GTR + I + G, GTR + I + G, and GTR + I + G, respectively.

The maximum-likelihood phylogenetic analysis was performed in RAxML v8.2.4 (Stamatakis, 2014). Given that this software has the constraint of using the same type of substitution model across all data subsets, we assigned a GTR + G model to each of the four subsets. Bootstrap support values were estimated using 1,000 pseudoreplicates. Two replicates of the analysis were performed to check for local optima, each using 10 random starts.

The phylogeny and divergence times were co-estimated using the Bayesian phylogenetic software BEAST v.1.8.4 (Drummond et al., 2012). To check for rate variation across branches, we ran analyses using a strict clock and an uncorrelated lognormal relaxed molecular clock (Drummond et al., 2006). To check the sensitivity of the results to the choice of tree prior, we conducted analyses using a Yule speciation model and using a birth-death model for the tree prior. All model comparisons were done using marginal likelihoods, calculated using the stepping-stone estimator (Xie et al., 2011). To examine the joint prior distribution of divergence times, we performed a BEAST analysis in which we drew samples from the prior (with the tree topology fixed to match the maximum-clade-credibility tree inferred using the sequence data).

Posterior distributions of parameters were estimated using Markov chain Monte Carlo (MCMC) sampling over a total of 10^8^ steps, with samples drawn every 10^4^ steps. The initial 10% of samples were discarded as burn-in. Convergence was checked by running the analysis in duplicate and by visualizing the results in the program Tracer v1.6 (Rambaut & Drummond, 2014), which showed that the effective sample sizes of all parameters were above 200. We used TreeAnnotator v1.8.4, part of the BEAST package, to identify the maximum-clade-credibility tree and to scale the node ages to the median posterior estimates.

### Fossil calibrations

Fossil evidence was used to constrain the ages of seven nodes in the tree (Table 1). We implemented each of these in the form of a uniform prior, which allows the node an equal probability of taking any age within the specified bounds (Ho & Phillips, 2009). Additionally, we performed an analysis using exponential priors. The first calibration point provided a maximum age for the separation of Stomatopoda from the other malacostracans. Phyllocarids are the oldest malacostracans in the fossil record, appearing in the Cambrian at least 500 Ma (Sepkoski, 1998; Collette & Hagadorn, 2010). Therefore, the maximum age constraint for the age of the root was set to the beginning of the Cambrian period (541 Ma).

**Table 1:** Calibration table. Fossil calibrations used in the molecular-clock analysis of the stomatopod evolutionary timescale. For each uniform prior, the minimum and maximum age constraints are given.

Fossil taxon	Higher classification	Node	Uniform prior (Ma)
*Daidal acanthocercus*^a^	Stomatopoda: Archaeostomatopodea	Stomatopoda vs *Homarus*	313–541
*Hemisquilla adelaidensis*^a^	Stomatopoda: Gonodactyloidea	*Hemisquilla* vs remaining Stomatopoda	11.6–313
*Lysiosquilla nkporoensis*^a^	Stomatopoda: Lysiosquilloidea	*Lysiosquillina* vs *Pullosquilla*	71–313
*Rhabdouraea bentzi*^b^	Leptostraca: Nebaliacea	*Paranebalia* vs all other taxa	259–541
*Neogonodactylus oerstedii*^a^	Stomatopoda: Gonodactyloidea	*Neogonodactylus* vs *Gonodactylus + Gonodactylellus*	11.6–313
*Pseudosquilla berica*^c^	Stomatopoda: Gonodactyloidea	*Pseudosquilla* vs *Raoulserenea*+* Pseudosquillana*	23–313
*Ursquilla yehoachi*^d^	Stomatopoda: Squilloidea	Squilloidea vs Parasquilloidea	72–313

**Notes.**

a Schram et al. (2013).

b Schram & Malzahn (1984).

c De Angeli & Messina (1996).

d Haug et al. (2013).

The second calibration point provided a minimum age for crown stomatopods. We note that a Devonian age for Palaeostomatopodea has been proposed based on interpretation of Eopteridae as probable palaeostomatopodeans (Schram et al., 2013). Given that Eopteridae is based on fragmentary fossils of uncertain identity, we take a more conservative approach and base our calibration on unambiguous hoplocarid fossils. This age was determined by the appearance of Palaeostomatopodea and the proto-stomatopods Archaeostomatopodea in the Carboniferous approximately 313 Ma, particularly the well-described *Daidal acanthocercus* fossil (Schram, 2007). These are regarded as representatives of the stem lineage leading to crown-group Stomatopoda.

Five calibration points were implemented as minimum bounds on the ages of unipeltatan stomatopod superfamilies, families, and genera. We selected reliable fossils of Gonodactyloidea, Lysiosquilloidea, and Squilloidea. The oldest putative gonodactyloid fossil, *Paleosquilla brevicoxa* from the Cretaceous, could not be used in our analysis. The incompleteness of *Paleosquilla brevicoxa* together with the non-monophyly of Gonodactyloidea, prevented a confident assignment of the fossil taxon to any particular gonodactyloid clade. However, three fossil calibrations were implemented for Gonodactyloidea. These provided minimum age constraints on extant genera within the families Gonodactylidae, Hemisquillidae, and Pseudosquillidae (Schram et al., 2013). One calibration for Lysiosquilloidea was selected on the basis of the sister relationship between *Lysiosquilla* and *Lysiosquillina* (Ahyong & Harling, 2000). The oldest squilloid fossil, *Ursquilla yehoachi*, which belongs to either the *Squilla* or *Oratosquilla* groups (Ahyong, 2005; Haug et al., 2013), was used to specify a minimum age constraint for the divergence between Squilloidea and Parasquilloidea. Finally, a minimum age constraint was placed on the root of the tree, representing the divergence between *Paranebalia* (Leptostraca) and all other taxa, based on the oldest known leptostracan fossil, *Rhabdouraea bentzi*, from the Upper Permian (Schram & Malzahn, 1984).

### Topology tests and ancestral state reconstruction

We used likelihood and Bayesian methods to test the monophyly of three groups: Gonodactyloidea, smashers, and smashers and spearers. To do this, we repeated the phylogenetic analyses in RAxML and BEAST as described above, but with the monophyly of each of the three groups being enforced in turn. The likelihood scores of the constrained and unconstrained tree topologies were compared using the approximately unbiased (AU) test in CONSEL (Shimodaira & Hasegawa, 2001). Bayesian tests of topology were done by calculating Bayes factors, based on marginal likelihoods calculated using the stepping-stone estimator in BEAST. Bayes factors were interpreted according to the recommendations of Kass & Raftery (1995).

Ancestral states were reconstructed using maximum likelihood to study the evolution of the advanced stomatopod eye. The number of midband rows and the shape of midband ommatidia were reconstructed at internal nodes in the tree using the program Mesquite v3.03 (Maddison & Maddison, 2015). Character states for modern taxa were recognized as categorical data and sourced from the literature (Ahyong, 1997; Ahyong, 2001; Porter et al., 2010). To trace the evolution of these visual characters, we used the maximum-clade-credibility tree from our Bayesian phylogenetic analysis, which was pruned using TreeGraph 2 (Stöver & Müller, 2010) to include only the species with known states for each of the characters.

**Figure 1:**
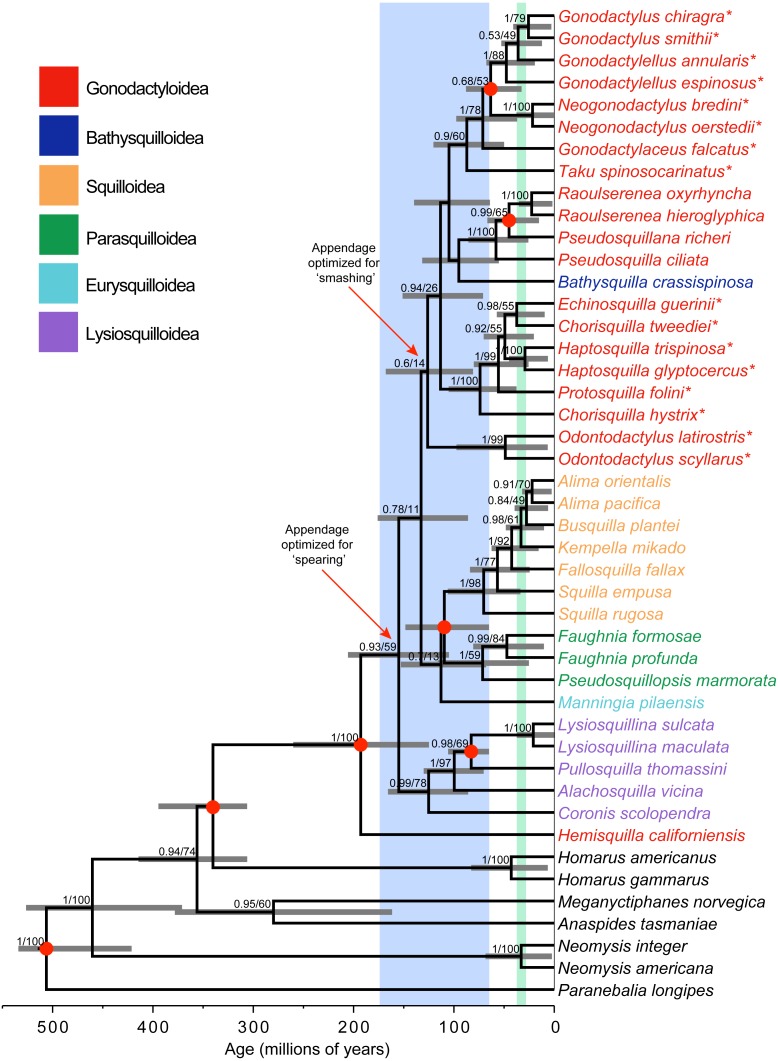
Phylogeny and divergence analysis. Bayesian estimate of stomatopod phylogeny, with branch lengths proportional to time scale. Blue shading corresponds to the breakup of the supercontinent Pangaea. Green shading corresponds to the major closing of the Tethys Sea. Support values (posterior probability and likelihood bootstrap support) are given for nodes with posterior probability >0.50. Grey horizontal bars denote 95% credibility intervals of estimates of node ages. Asterisks (*) indicate ‘smashing’ stomatopods. Red dots on nodes correspond to fossil calibrations.

## Results

### Stomatopod phylogeny

Bayesian and maximum-likelihood analyses of the three mitochondrial and two nuclear markers yielded almost identical estimates of the stomatopod phylogeny, although with differing levels of node support (Fig. 1). *Hemisquilla* was consistently placed as the sister group to all other extant stomatopods. The analyses yielded moderate to low support for nodes along the backbone of the tree. Each superfamily was inferred to be monophyletic with high posterior probability, with the exception of a polyphyletic Gonodactyloidea. Smashing stomatopods were placed in a relatively nested position within the tree. Although smashing had a monophyletic origin, a clade containing spearers, Pseudosquillidae and Bathysquillidae, was nested among the smashers (albeit with weak support).

Five of the six stomatopod superfamilies included in our study were found to be monophyletic, the exception being the polyphyletic Gonodactyloidea (Fig. 1). Topology tests were used to evaluate support for the monophyly of three groups: Gonodactyloidea (with and without *Hemisquilla*), smashers, and smashers and spearers. The AU test failed to reject the hypothesis of monophyly in all four cases (*P* > 0.05; Table 2). In contrast, the Bayesian analyses consistently favoured the unconstrained tree topology over the constrained topologies, except with respect to monophyletic smashers and spearers, with log Bayes factors ranging from −2015.6 to 4346.7 (Table 2). We note that this result has been obtained even though Bayes factors tend to be biased towards the hypotheses involving constrained tree topologies (Bergsten, Nilsson & Ronquist, 2013).

**Table 2:** Topology test table. Tests of the monophyly of four different clades in the stomatopod phylogeny.

Tree	*P*-value^a^ (AU test)	Bayesian	
		Marginal ln(L)^b^	ln(BF)^c^
Unconstrained		−44,590.7	
Monophyletic Gonodactyloidea	0.417	−46,746.2	2155.5
Monophyletic Gonodactyloidea (excl. *Hemisquilla*)	0.264	−46,001.0	1410.3
Monophyletic smashers	0.411	−48,937.4	4346.7
Monophyletic smashers and spearers	0.360	−42,575.1	−2015.6

**Notes.**

a *P*-value from an approximately unbiased test against the unconstrained tree. *P* < 0.05 indicates that the topology constraint should be rejected.

b Marginal log likelihood estimated using stepping-stone sampling.

c Log Bayes factor comparing unconstrained against the constrained topology. Values greater than 3 indicate strong support, whereas values greater than 5 indicate very strong support (Kass & Raftery, 1995).

### Estimates of divergence times

Comparison of marginal likelihoods revealed strong support for an uncorrelated lognormal relaxed clock (lnL = − 28, 550.8) over a strict clock (lnL = − 28, 668.3), indicating the presence of rate variation across branches. In addition, we found support for a Yule tree model (lnL = − 28, 550.8) over a birth-death model (lnL = − 28, 556.0). The choice of tree prior did not have a measurable impact on the resulting estimates of divergence times. Furthermore, the marginal prior distribution of node times differed from the posterior estimates, indicating the presence of an informative evolutionary signal in the sequence data (Fig. S1).

Stomatopoda diverged from its closest crustacean relatives 340 Ma (95% CRI [401–313 Ma]; Fig. 1). The crown-group unipeltatan stomatopods arose 193 Ma (95% CRI [267–131 Ma]), when the lineage leading to *Hemisquilla* split from remaining stomatopods. Squilloidea was found to be a relatively young superfamily, with the first divergence within the group occurring 70 Ma (95% CRI [113–40 Ma]). Stomatopods with specialized spearing claws arose about 155 Ma (95% CRI [212–111 Ma]), with specialized smashers appearing 126 Ma (95% CRI [174–87 Ma]).

### Ancestral state reconstructions

**Figure 2:**
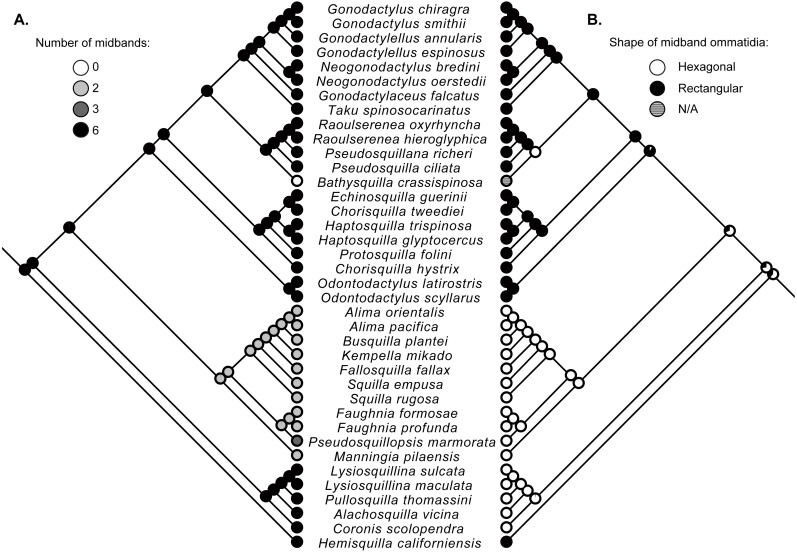
Ancestral state reconstructions. Maximum-likelihood ancestral state reconstructions of the number of midband rows (A) and the shape of midband ommatidia (B) within the stomatopod eye based on a subset of taxa from the estimate of the stomatopod phylogeny (Fig. 1). Proportional likelihood of each character is represented by the pie chart at each internal node.

Character state reconstructions indicate that the ancestral adult stomatopod eye contained six midband rows and hexagonal ommatidia (Fig. 2). Species in two superfamilies (Gonodactyloidea and Lysiosquilloidea) in this study possess six midbands, whereas the remaining superfamilies have lost complexity in this character (Fig. 2). The parasquilloid, *Pseudosquillopsis,* has lost three midbands, while *Faughnia* has lost four, leaving two midbands. Similarly, species in Squilloidea and some in Eurysquilloidea have lost four midbands, whereas bathysquilloids have lost all six midbands. Gonodactyloidea is the only superfamily with rectangular midband ommatidia; all other superfamilies have hexagonal midband ommatidia. The ancestral state reconstructions suggest that rectangular midband ommatidia evolved independently in Hemisquillidae and in the remaining Gonodactyloidea.

## Discussion

### Phylogenetic relationships

This study presents the most comprehensive molecular phylogenetic analysis of Stomatopoda so far, based on representatives from six superfamilies. Our estimates of stomatopod relationships are congruent with the findings of Porter et al. (2010), who inferred a weakly supported sister relationship between Gonodactyloidea and Squilloidea using four markers (*16S*, *18S*, *CO1*, and *28S*). In contrast, Ahyong & Jarman (2009) found Lysiosquilloidea and Squilloidea to be sister clades, based on an analysis of three markers (*12S*, *16S*, and *28S*). Our analysis is the first to include representatives from the superfamilies Eurysquilloidea and Bathysquilloidea. The eurysquilloid representative is the sister lineage to a clade comprising Squilloidea and Parasquilloidea, which is consistent with its superfamily status. Additionally, the close relationship between Eurysquilloidea, Parasquilloidea, and Squilloidea is consistent with evidence from their morphology (Ahyong & Harling, 2000; Ahyong, 2001; Ahyong, 2005). However, our analysis placed the single bathysquilloid as the sister taxon to Pseudosquillidae, a position that is not supported by morphological evidence.

The placement of *Hemisquilla* as the sister lineage to all other stomatopods corroborates the results of previous molecular studies by Ahyong & Jarman (2009) and Porter et al. (2010). Collectively, these results support the hypothesis that hemisquillids are not a true gonodactyloid lineage. This suggests that Hemisquillidae should be moved to a separate superfamily (Porter et al., 2010), although such an action would be premature until rare morphologically intermediate taxa (Alainosquillidae) can be analysed. The phylogenetic placement of *Hemisquilla* is consistent with its seemingly plesiomorphic morphology, such as its subcylindrical body, and raptorial appendage that is neither highly optimized for powerful smashing nor efficient spearing, closely resembling that of the stem-lineage pseudosculdids (Ahyong & Harling, 2000; Ahyong, Garassino & Gironi, 2007; Ahyong & Jarman, 2009). Additionally, the preference of *Hemisquilla* for soft substrates is more similar to that of squilloids and other spearers than to gonodactyloids, which usually occupy hard substrate cavities (Schram et al., 2013).

The composition of the remaining Gonodactyloidea (apart from the anomalous inclusion of *Bathysquilla*, which represents a distinct superfamily) is congruent with previous molecular and morphological evidence, corroborating the removal of Eurysquillidae and Parasquillidae to separate superfamilies by Ahyong & Harling (2000). Additionally, the inferred placement of Pseudosquillidae within Gonodactyloidea in our study accords with morphological evidence (Ahyong & Harling, 2000) and differs from the near-basal position among stomatopods (albeit weakly supported) inferred by Ahyong & Jarman (2009) and Porter et al. (2010). However, the Bayesian topology test suggests a non-monophyletic Gonodactyloidea (excluding *Hemisquilla*), indicating that further exclusions might be necessary. The inferred placement of Odontodactylidae within Gonodactyloidea is consistent with those of both Porter et al. (2010) and Barber & Erdmann (2000), who estimated Odontodactylidae to be the sister group to all other smashing gonodactyloids. Such a result is consistent with morphological evidence and a monophyletic smashing clade (Ahyong & Harling, 2000).

### Evolutionary timescale of stomatopods

Our molecular-clock analysis has provided an estimate of when the major stomatopod superfamilies diverged, shedding light on the geological events that might have been associated with the diversification of the order. The results of our analyses support fossil evidence for a Carboniferous divergence between Stomatopoda and other malacostracans (Ahyong, 1997; Hof, 1998; Ahyong & Jarman, 2009).

The estimates of divergence times are consistent with the notion that the break-up of the supercontinent Pangaea was associated with a key period in the evolution of Stomatopoda, which has been suggested previously for *Hemisquilla* (Schram, 1977; Schram et al., 2013). Diversification of extant stomatopod lineages can be traced to the beginnings of the break-up of Pangaea (i.e., ∼175 Ma; Fig. 1), with all of the divergences among superfamilies occurring from 193 to 95 Ma. Diversification might have coincided with the creation of new coastal habitats, which became available as landmasses were separated to form the Tethys Sea. As mentioned above, stomatopods predominantly occur in tropical coastal waters; therefore, the appearance of new habitats might have allowed the group to expand substantially.

The diversification of stomatopods with the opening of new habitats is consistent with findings from other crustaceans, especially decapods (George, 2006; Palero et al., 2009). George (2006) proposed that spiny lobsters (Palinuridae) radiated to new habitats as continental plates shifted, fragmenting the Tethys Sea and dividing Southern Ocean habitats. The age estimates from our analysis, along with the largely cosmopolitan distribution of stomatopod families and superfamilies, indicate that these lineages probably originated in the Tethys Sea when sea levels were high during the Cretaceous (Veron, 1995; George, 2006). The major superfamilies diverged before the closing of the seaway during the Paleogene/Neogene and the subsequent separation of the Atlantic and Pacific Oceans (Ekman, 1953; Reaka, Rodgers & Kudla, 2008; Schram et al., 2013).

Both Bathysquilloidea and Squilloidea have previously been suggested as “basal” lineages in the Unipeltata. These hypotheses were based on superficial morphological similarities with the extinct sculdids in the segmentation of the uropodal exopod in the bathysquilloid *Indosquilla*, and strong dorsal carination of squillids, said to somewhat resemble that of the Jurassic sculdids (Kemp, 1913; Manning, Kropp & Dominguez, 1990). Our analysis supports a “basal” position for neither Bathysquilloidea nor Squilloidea, instead suggesting that Hemisquillidae is likely to be the sister lineage to all other stomatopods, which is supported by morphological and other molecular evidence (Ahyong & Jarman, 2009; Schram et al., 2013). However, because only a single hemisquillid was included in the analysis, the results require further corroboration using additional exemplars.

### Evolution of raptorial appendages and vision

Two hypotheses regarding the origin of the raptorial appendage have been proposed. The first suggests that specialized smashing evolved from within the spearers after a long history of specialized spearing (Caldwell, 1991; Ahyong, 1997). The second proposes that specialized smashing and specialized spearing evolved early in the history of Unipeltata, with more-or-less monophyletic spearing and smashing clades, perhaps diverging from a *Hemisquilla*-like ancestor (Ahyong & Harling, 2000; Ahyong & Jarman, 2009). Our results reprise aspects of both hypotheses, being similar to the first in finding smashers deeply nested among the spearers. However, our analysis infers a short branch on the ancestral node leading to the smashers. This indicates that smashing evolved relatively quickly and might also account for the weak molecular support for the grouping of odontodactylids with other smashers, at least among the markers used in this study. Porter et al. (2010) also recovered a similarly short branch leading to the smashers. Moreover, a link has been suggested between the evolution of the smashing appendage and the diversification of coral reefs in the Cretaceous; this could explain the rapid diversification of Gonodactyloidea during this time (Ahyong & Harling, 2000). Owing to the weak support for a monophyletic Gonodactyloidea, further molecular research into the boundaries of the superfamily is clearly required.

Ancestral state reconstructions of the complex stomatopod visual systems suggest that the eyes of stem-lineage unipeltatans had six midbands as also found by Porter et al. (2010), but with hexagonal rather than rectangular ommatidia. Groups, such as squilloids, parasquilloids, bathysquilloids and some eurysquilloids, with fewer or no midbands, are deeply nested within the crown-group. This suggests that the reduction in morphological complexity occurred independently in different lineages. This reduction in complexity might be linked to the visual environment, which is strongly influenced by depth and turbidity (Schram et al., 2013).

Further support for the influence of environment can be seen in the eyes of bathysquilloids, parasquilloids, and squilloids, which have varying degrees of ommatidial reduction. These groups inhabit deep or turbid waters and reduced eyes and loss of visual complexity are typical adaptations to these environments (MacDonald, 1975; Gaten, 1998; Lins et al., 2012). Additionally, squilloids and possibly parasquilloids are thought to have monochromatic vision, having lost some or all bands for colour (parasquilloids variously have two or three ommatidial midbands; Cronin, 1985; Ahyong, 2005); further investigation is needed to confirm this. Monochromatic vision is advantageous in the turbid, dark waters that parasquilloids and squilloids usually inhabit, where increased sensitivity to movement of prey and predators is more important than colour resolution. Bathysquilloids, living at great depth, have not only lost all ommatidial midbands, but the ommatidia on the cornea surface are themselves highly reduced.

## Conclusions

Our study provides the most extensive molecular phylogenetic analysis of the Stomatopoda. Although the relationships among superfamilies are only resolved with moderate confidence, the monophyly of most superfamilies is well supported. The exception to this is the superfamily Gonodactyloidea, in which Bathysquilloidea is nested and from which Hemisquillidae is excluded. Our molecular estimates of the evolutionary timescale indicate that the extant superfamilies possibly diverged from each other during the formation of the Tethys Sea in the early Cretaceous (∼175 Ma), and that the specialized smashing claw evolved in the late Cretaceous (∼126 Ma). Although our analysis provides insights into the evolutionary timescale of Stomatopoda, it also highlights how much is still unknown. Our study draws attention to possible directions for future work on the group, particularly with the aim of resolving the boundaries, relationships, and origins of the extant superfamilies.

##  Supplemental Information

10.7717/peerj.3844/supp-1 Figure S1 Supplementary sampling from the prior figure A. Joint prior distribution of divergence times for the stomatopod tree, with blue bars representing the 95% highest prior density intervals. B. Posterior distribution of divergence times, with blue bars representing the 95% highest posterior density intervals (fixed topology based on Fig. 1). C. Posterior distribution of divergence times using exponential priors, with blue bars representing the 95% highest posterior density intervals. Blue lines join the same node between trees.

10.7717/peerj.3844/supp-2 Table S1 Taxa Table List of stomatopod and outgroup taxa, along with GenBank accession numbers, included in this study. Dash (-) indicates missing sequence. *28S-A* includes the D2-D7B region, while *28S-B* includes the D9-D10 regions.

10.7717/peerj.3844/supp-3 Table S2 Primers Table Primers used in this study to amplify regions of the *12S*, *16S*, and 28S-D1 segments of the stomatopod mitochondrial and nuclear genomes.

10.7717/peerj.3844/supp-4 Table S3 Saturation test results table Results of Xia’s saturation test in DAMBE6 for each of the markers analysed in this study. Values are based on random 32-taxon subsamples of the complete data set.

10.7717/peerj.3844/supp-5 Supplemental Information 1 Genbank Submission File For review only.

## Additional Information and Declarations

### DNA Deposition

The following information was supplied regarding the deposition of DNA sequences: DNA sequences are available on GenBank. Accession numbers KY236044–KY236055 and KY436058–KY436061.

### Data Availability

The following information was supplied regarding data availability: GitHub: https://github.com/caravanderwal/Stomatopoda/.
