# Supplementary figures and images for: The evolutionary history of Stomatopoda (Crustacea: Malacostraca) inferred from molecular data
Source: PeerJ. 2017 Sep 21;5:e3844. doi: 10.7717/peerj.3844 (PMC5610894; doi:10.7717/peerj.3844)

**A.**

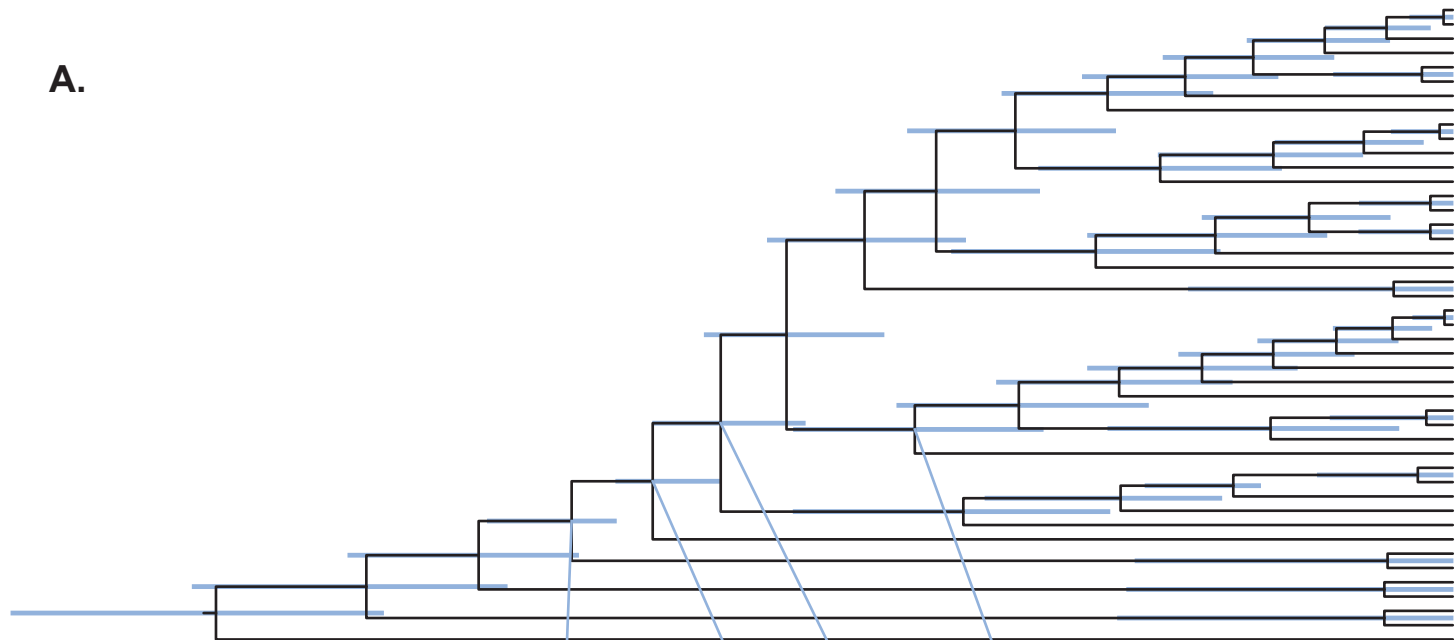

**B.**

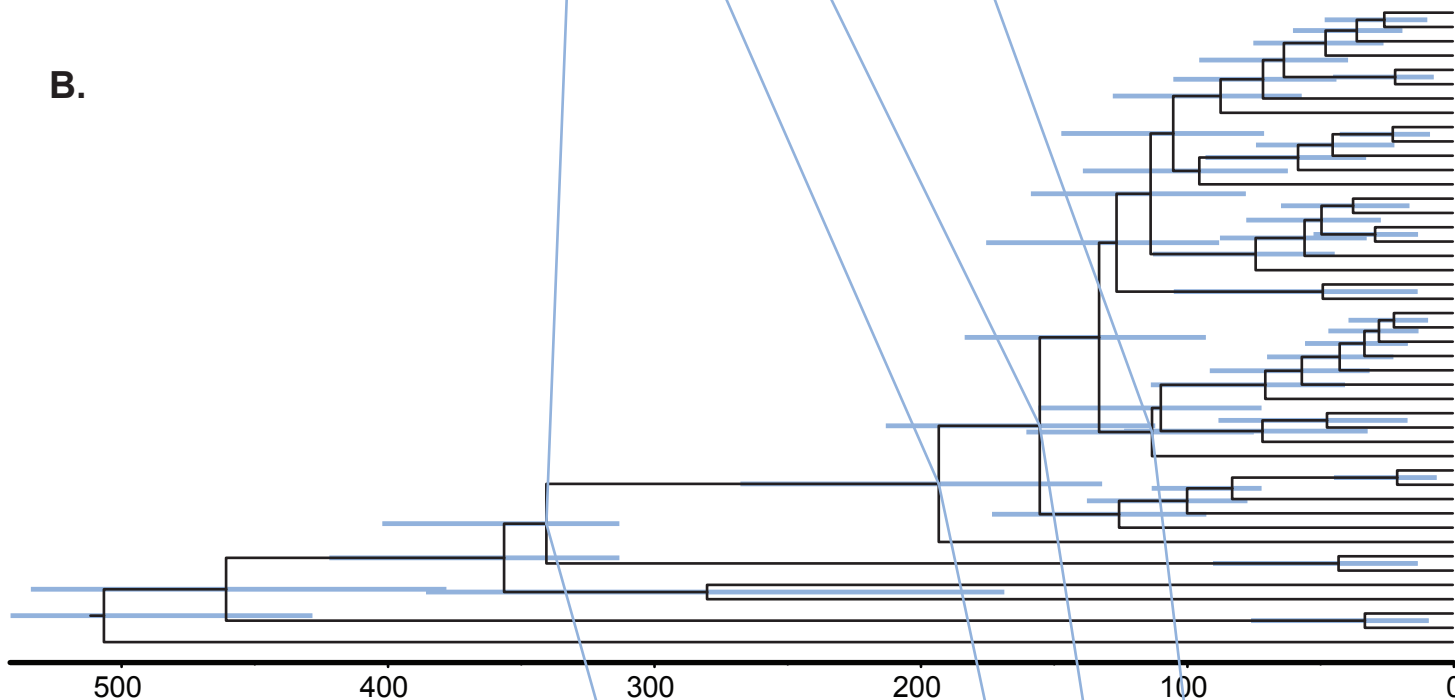

**C.**

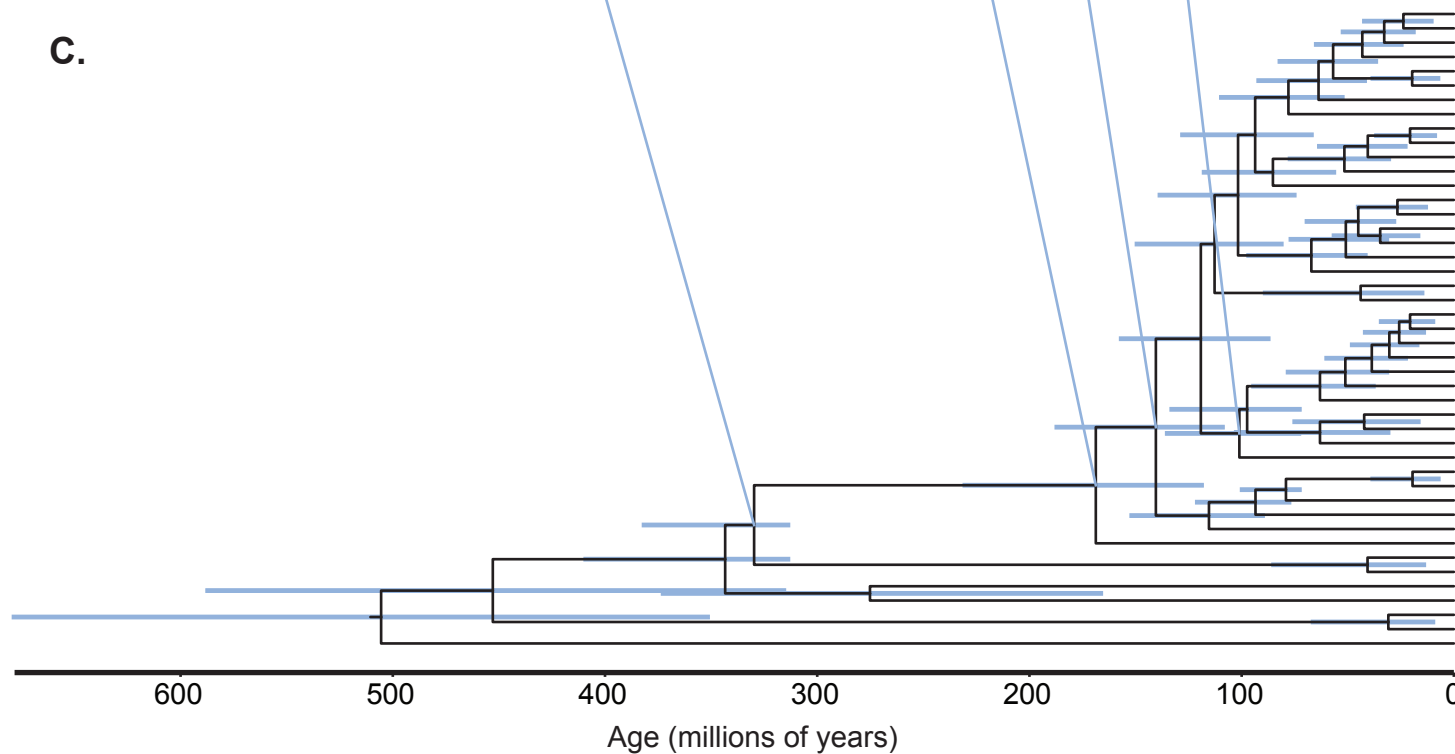

Supplement: Figure S1 — A. Joint prior distribution of divergence times for the stomatopod tree, with blue bars representing the 95% highest prior density intervals. B. Posterior distribution of divergence times, with blue bars representing the 95% highest posterior density intervals (fixed topology based on Fig. 1). C. Posterior distribution of divergence times using exponential priors, with blue bars representing the 95% highest posterior density intervals. Blue lines join the same node between trees. [file peerj-05-3844-s001.pdf]
