# Supplementary material for: The evolutionary history of Stomatopoda (Crustacea: Malacostraca) inferred from molecular data
Source: PeerJ. 2017 Sep 21;5:e3844. doi: 10.7717/peerj.3844 (PMC5610894; doi:10.7717/peerj.3844)
Supplement: Table S1 — List of stomatopod and outgroup taxa, along with GenBank accession numbers, included in this study. Dash (-) indicates missing sequence. 28S-A includes the D2-D7B region, while 28S-B includes the D9-D10 regions. [file peerj-05-3844-s002.docx]

| **Taxon** | ***12S*** | ***16S*** | ***18S*** | ***CO1*** | ***28S-A*** | ***28S-B*** |
| --- | --- | --- | --- | --- | --- | --- |
| **Bathysquilloidea** |  |  |  |  |  |  |
| **Bathysquillidae** |  |  |  |  |  |  |
| *Bathysquilla crassispinosa* | KY236050 | KY236045 | - | - | KY236053 | - |
| **Eurysquilloidea** |  |  |  |  |  |  |
| **Eurysquillidae** |  |  |  |  |  |  |
| *Manningia pilaensis* | KY236051 | KY236044 | - | - | KY236054 | - |
| **Gonodactyloidea** |  |  |  |  |  |  |
| **Gonodactylidae** |  |  |  |  |  |  |
| *Gonodactylaceus falcatus* | - | HM138827 | HM138871 | HM138786 | HM180015 | HM180059 |
| *Gonodactylellus annularis* | - | HM138824 | HM138868 | HM138783 | HM180012 | HM180056 |
| *Gonodactylellus espinosus* | - | HM138822 | HM138866 | HM138782 | HM180010 | HM180054 |
| *Gonodactylus chiragra* | AF107594 | HM138826 | HM138870 | HM138785 | HM180014 | HM180058 |
| *Gonodactylus smithii* | AF107595 | HM138829 | HM138873 | HM138788 | HM180017 | HM180061 |
| *Neogonodactylus bredini* | - | HM138837 | HM138866 | HM138795 | HM180025 | HM180069 |
| *Neogonodactylus oerstedii* | AF107596 | HM138838 | HM138882 | HM138796 | HM180026 | HM180070 |
| **Hemisquillidae** |  |  |  |  |  |  |
| *Hemisquilla californiensis* | - | HM138832 | HM138876 | HM138791 | HM180020 | HM180064 |
| **Odontodactylidae** |  |  |  |  |  |  |
| *Odontodactylus latirostris* | - | HM138841 | HM138885 | HM138797 | HM180029 | HM180073 |
| *Odontodactylus scyllarus* | - | HM138842 | HM138886 | HM138798 | HM180030 | HM180074 |
| **Protosquillidae** |  |  |  |  |  |  |
| *Chorisquilla hystrix* | - | HM138817 | HM138861 | HM138777 | HM180005 | HM180049 |
| *Chorisquilla tweediei* | - | HM138818 | HM138862 | HM138778 | HM180006 | HM180050 |
| *Echinosquilla guerinii* | - | HM138820 | HM138864 | HM138780 | HM180008 | HM180052 |
| *Haptosquilla glyptocercus* | AF107599 | HM138830 | HM138874 | HM138789 | HM180018 | HM180062 |
| *Haptosquilla trispinosa* | - | HM138831 | HM138875 | HM138790 | HM180019 | HM180063 |
| *Protosquilla folini* | - | HM138843 | HM138887 | HM138799 | HM180031 | HM180075 |
| **Pseudosquillidae** |  |  |  |  |  |  |
| *Pseudosquilla ciliata* | AY947836 | HM138844 | HM138888 | HM138800 | HM180032 | HM180076 |
| *Pseudosquilliana richeri* | - | HM138846 | HM138890 | HM138802 | HM180034 | HM180078 |
| *Raoulserenea hieroglyphica* | - | HM138848 | HM138892 | HM138805 | HM180037 | HM180081 |
| *Raoulserenea oxyrhyncha* | - | HM138851 | HM138895 | HM138807 | HM180039 | HM180083 |
| **Takuidae** |  |  |  |  |  |  |
| *Taku spinosocarinatus* | AF107600 | HM138855 | HM138899 | HM138811 | HM180043 | HM180087 |
| **Lysiosquilloidea** |  |  |  |  |  |  |
| **Lysiosquillidae** |  |  |  |  |  |  |
| *Lysiosquillina maculata* | AF107603 | HM138834 | HM138878 | HM138793 | HM180022 | HM180066 |
| *Lysiosquillina sulcata* | - | HM138835 | HM138879 | - | HM180023 | HM180067 |
| **Nannosquillidae** |  |  |  |  |  |  |
| *Alachosquilla vicina* | AF107601 | HM138812 | HM138856 | - | HM180000 | HM180044 |
| *Coronis scolopendra* | - | HM138819 | HM138863 | HM138779 | HM180007 | HM180051 |
| *Pullosquilla thomassini* |  | HM138847 | HM138891 | HM138803 | HM180035 | HM180079 |
| **Parasquilloidea** |  |  |  |  |  |  |
| **Parasquillidae** |  |  |  |  |  |  |
| *Faughnia formosae* | KY236048 | KY236047 | - | - | KY236055 | - |
| *Faughnia profunda* | KY236049 | KY236046 | - | - | KY236052 | - |
| *Pseudosquillopsis marmorata* | - | HM138845 | HM138889 | HM138801 | HM180033 | HM180077 |
| **Squilloidea** |  |  |  |  |  |  |
| **Squillidae** |  |  |  |  |  |  |
| *Alima orientalis* | KY436058 | HM138813 | HM138857 | HM138773 | HM180001 | HM180045 |
| *Alima pacifica* | KY436059 | HM138814 | HM138858 | HM138774 | HM180002 | HM180046 |
| *Busquilla plantei* | KY436060 | HM138815 | HM138859 | HM138775 | HM180003 | HM180047 |
| *Fallosquilla fallax* | - | HM138821 | HM138865 | HM138781 | HM180009 | HM180053 |
| *Kempella mikado* | KY436061 | HM138833 | HM138877 | HM138792 | HM180021 | HM180065 |
| *Squilla empusa* | AF107605 | HM138853 | HM138897 | HM138809 | HM180041 | HM180085 |
| *Squilla rugosa* | - | HM138854 | HM138898 | HM138810 | HM180042 | HM180086 |
| **Outgroups** |  |  |  |  |  |  |
| *Anaspides tasmaniae* | - | AF133694 | L81948 | DQ889076 | AY859549 | - |
| *Homarus americanus* | DQ298427 | AF370876 | AY743945 | AF370853 | DQ079788 | - |
| *Homarus gammarus* | NC_020020 | NC_020020 | DQ079749 | EU186145 | DQ079789 | - |
| *Meganyctiphanes norvegica* | - | AY744910 | DQ900731 | AF177191 | AY744900 | - |
| *Neomysis americana* | - | HM179997 | HM179998 | FJ581789 | HM179999 | - |
| *Neomysis integer* | - | FJ374668 | AJ566091 | AY529029 | - | - |
| *Paranebalia longipes* | - | AY744909 | EF189630 | - | EF189655 | - |
