# Supplementary material for: The evolutionary history of Stomatopoda (Crustacea: Malacostraca) inferred from molecular data
Source: PeerJ. 2017 Sep 21;5:e3844. doi: 10.7717/peerj.3844 (PMC5610894; doi:10.7717/peerj.3844)
Supplement: Table S2 — Primers used in this study to amplify regions of the 12S, 16S, and 28S-D1 segments of the stomatopod mitochondrial and nuclear genomes. [file peerj-05-3844-s003.docx]

| **Gene** | **Direction** | **Name** | **Sequence (5’ - 3’)** | **Reference** |
| --- | --- | --- | --- | --- |
| *12S* | Forward |  | GAAACCAGGATTAGATACCC | Mokady & Brickner (2001); Mokady et al. (1994) |
| *12S* | Reverse |  | TTTCCCGCGAGCGACGGGCG | Mokady & Brickner (2001); Mokady et al. (1994) |
| *16S* | Forward | 16Sar-L | CGCCTGTTTATCAAAAACAT | Ahyong & Jarman (2009) |
| *16S* | Reverse | 16Sbr-H | CCGGTCTGAACTCAGATCACGT | Ahyong & Jarman (2009) |
| *28S* | Forward | 28S-F216 | CTGAATTTAAGCATATTAATTAGKGSAGG | Ahyong et al. (2009); Schnabel et al. (2011) |
| *28S* | Reverse | 28S-R443 | CCGATAGCGAACAAGTACCGTGAGG | Ahyong et al. (2009); Schnabel et al. (2011) |

**References**

Ahyong, S.T., Jarman, S.N., 2009. Stomatopod interrelationships: Preliminary results based on analysis of three molecular loci. Arthropod Syst. Phylo. 67, 91–98.

Ahyong, S.T., Schnabel, K.E., Maas, E., 2009. Anomuran phylogeny: New insights from molecular data. Crustac. Issues. 18: Decapod Crustacean Phylogenetics (ed. By J.W. Martin, K.A. Crandall and D.L. Felder). CRC Press, Boca Raton.

Mokady, O., Brickner, I., 2001. Host-associated speciation in a coral-inhabiting barnacle. Mol. Biol. Evol. 18, 975–981.

Mokady, O., Rozenblatt, S., Graur, D., Loya, Y., 1994. Coral-host specifity of Red Sea *Lithophaga* bivalves: Interspecific and intraspecific variation in 12S mitochondrial ribosomal RNA. Mol. Mar. Biol. Biotech. 3, 158–164.

Schnabel, K.E., Ahyong, S.T., Maas, E.W., 2011. Galatheoidea are not monophyletic - molecular and morphological phylogeny of the squat lobsters (Decapoda: Anomura) with recognition of a new superfamily. Mol. Phylogenet. Evol. 58, 157–168.
