# Supplementary material for: The evolutionary history of Stomatopoda (Crustacea: Malacostraca) inferred from molecular data
Source: PeerJ. 2017 Sep 21;5:e3844. doi: 10.7717/peerj.3844 (PMC5610894; doi:10.7717/peerj.3844)
Supplement: Table S3 — Results of Xia’s saturation test in DAMBE6 for each of the markers analysed in this study. Values are based on random 32-taxon subsamples of the complete data set. [file peerj-05-3844-s004.docx]

| **Gene** | **Symmetrical tree (P-value)** | **Asymmetrical tree (P-value)** |
| --- | --- | --- |
| *12S* | 0.000 | 0.000 |
| *16S* | 0.000 | 0.0035 |
| *18S* | 0.000 | 0.000 |
| *28S* | 0.000 | 0.000 |
| *CO1* (1^st^ codon position) | 0.000 | 0.000 |
| *CO1* (2^nd^ codon position) | 0.000 | 0.000 |
| *CO1* (3^rd^ codon position) | 0.6667 | 0.000 |
